# Analysis of the complete *Fischoederius elongatus* (Paramphistomidae, Trematoda) mitochondrial genome

**DOI:** 10.1186/s13071-015-0893-3

**Published:** 2015-05-20

**Authors:** Xin Yang, Yunyang Zhao, Lixia Wang, Hanli Feng, Li Tan, Weiqiang Lei, Pengfei Zhao, Min Hu, Rui Fang

**Affiliations:** State Key Laboratory of Agricultural Microbiology, College of Veterinary Medicine, Huazhong Agricultural University, Wuhan, 430070 Hubei People’s Republic of China; Hubei Provincial Center for Diseases Control and Prevention, Wuhan, 430079 Hubei People’s Republic of China; Hubei Entry-Exit Inspection and Quarantine Bureau, Wuhan, 430022 Hubei People’s Republic of China

**Keywords:** *Fischoederius elongates*, Mitochondrial genome

## Abstract

**Background:**

*Fischoederius elongates* is an important trematode of Paramphistomes in ruminants. Animals infected with *F. elongates* often don’t show obvious symptoms, so it is easy to be ignored. However it can cause severe economic losses to the breeding industry. Knowledge of the mitochondrial genome of *F. elongates* can be used for phylogenetic and epidemiological studies.

**Findings:**

The complete mt genome sequence of *F. elongates* is 14,120 bp in length and contains 12 protein-coding genes, 22 tRNA genes, two rRNA genes and two non-coding regions (LNR and SNR). The gene arrangement of *F. elongates* is the same as other trematodes, such as *Fasciola hepatica* and *Paramphistomum cervi*. Phylogenetic analyses using concatenated amino acid sequences of the 12 protein-coding genes by Maximum-likelihood and Neighbor-joining analysis method showed that *F. elongates* was closely related to *P. cervi*.

**Conclusion:**

The complete mt genome sequence of *F. elongates* should provide information for phylogenetic and epidemiological studies for *F. elongates* and the family Paramphistomidae.

## Findings

### Background

Paramphistomes are distributed worldwide and have been reported in many countries, such as Bulgaria, France, Poland, Hungary, Italy, India, Russia, Sardinia and Yugoslavia [1]. The paramphistome can infect fishes, reptiles, birds and mammals, some of which can lead to huge economic losses related to seriously gastrointestinal diseases, low producitivity or death in ruminants [2]. In Arumeru District, the prevalence rate of paramphistomes is as high as 56.7 % in cattle [3].

*Fischoederius elongates* is an important member of paramphistomes, the parasite usually inhabits the rumen of cattle, buffaloes, sheep and goats. Ruminants are usually infected by ingesting snails, such as *Lymnaea acuminata*, *Lymnaea succinea* or *Gyraulus euphraticus* [4]. Ruminants infected with *F. elongates* show weakness, mental fatigue and eventually death. More seriously, *F. elongates* maybe a zoonotic trematode, a Chinese woman from Guangdong Province was reported to be the first human infection case [5], but it is still unknown how she was infected.

Untill now, the most common diagnostic method for *F. elongates* is the microscopical examination, but it’s time-consuming, and hard to distinguish with other paramphistomes. As a useful marker, mt genome has been widely used for species identification [6–10]. The complete mt genome of *F. elongates* can provide alternative molecular markers for the species identification, epidemiology and genetic diversity of paramphistomes.

In the present study, we got the full sequence and gene arrangement of mt genome of *F. elongates* and compared it with selected trematodes. We found that *F. elongates* had the closest relationship with *P. cervi*.

## Methods

### Ethical approval

The study was performed under the instructions and approval of Laboratory Animals Research Centre of Hubei province in P. R. China and the ethics committee of Huazhong Agricultural University (Permit number: 4200695757).

### Parasite collection and DNA isolation

*F. elongates* adults were collected from the rumen and reticulum of naturally infected cattle in Zhanggang, Tianmen, Hubei province, PR China, according to the Animal Ethics Guidelines of Huazhong Agricultural University. Then, the adult worms were washed extensively in 0.9 % sodium chloride solution, and identified through morphological examinations [2]. Subsequently, one worm was stained for identification [11], and the rest were fixed in 75 % alcohol (V/V) and stored at −20 °C until use [12]. Total genomic DNA was isolated from one worm [13]. The ITS-2 region of *F. elongates* was amplified and sequenced as reported previously [14], it was 100 % similar to that of *F. elongates* (GenBank accession no. JQ688410.1).

### Amplification and sequencing of *F. elongates* mt genome

Firstly, we designed 12 oligonucleotide primers according to the conserved regions from reported mt genome sequences of *F. hepatica* [15], *Clonorchis sinensis* [16] and *P. cervi* [17] to amplify partial fragments from *cox*3, *cyt*b, *nad*4, *cox*1, *rrn*S and *nad*5 (Table 1). PCRs (25 μl) were performed in the following reaction: 10 mM Tris–HCl (pH 8.4), 50 mM KCl, 4 mM MgCl_2_, 200 mM each of dNTP, 50 pmol of each primer,2 U *Taq* polymerase (Takara) and 2.5 μl genomic DNA. Reactions were run under the following conditions: 94 °C for 5 min, followed by 35 cycles of 94 °C/30 s, 50 °C/30 s and 72 °C/1 min. Amplicons were sent to Sangon Company (Shanghai, China) for sequencing.

**Table 1:** Primers used in the present study

Primer codes	Sequences (5′-3′)	Target gene	References
XCCOX3F	AGYACDGTDGGDTTRCATTT	*cox*3^1^	Present study
XCCOX3R	CANAYATAATCMACARAATGNCA	*cox*3^1^	Present study
nxccobF	ATGTCWTWTTGRGCKGCBACNGT	*cyt*b^1^	Present study
nxccobR	GADVCTCNGGRTGRCAVGCHCC	*cyt*b^1^	Present study
nxcND4F	GAKTCBCCDTATTCDGARCG	*nad*4^1^	Present study
nxcND4R	ACHCCNGCHGANANMCCRTGMCC	*nad*4^1^	Present study
TXCCOX1F	GGHTGAACHRTWTAYCCHCC	*cox*1^1^	Present study
TXCCOX1R	TGRTGRGCYCAWACDAYAMAHCC	*cox*1^1^	Present study
XC12SF	AAWAAYGAGAGYGACGGGCG	*rrn*S^1^	Present study
XC12SR	TARACTAGGATTAGATACCC	*rrn*S^1^	Present study
NxcND5F	TGKTTGCBTCNCGNTTBGGNGATG	*nad*5^1^	Present study
NxcND5R	TAACACTTRCANAHMCCRTGHGT	*nad*5^1^	Present study
3CF1	TGCATGTAGTGATAGGTTTGG	*cox*3*- cyt*b^2^	Present study
3CR1	AACTAACGTAACATTTGTCAC	*cox*3*- cyt*b^2^	Present study
3CF2	TTTGTTTTGTGGTTGCCTTC	*cyt*b*-nad*4^2^	Present study
3CR2	AACGTAAATTAAACCTCCCCC	*cyt*b*-nad*4^2^	Present study
3CF3	TGGCGTTTTTGAGTTTGTCTC	*nad*4*-cox*1^2^	Present study
3CR3	TCAACGAACTCAATATACTTG	*nad*4*-cox*1^2^	Present study
3CF4	TGGTTTCGGGGCTGTGAGAC	*cox*1*-rrn*S^2^	Present study
3CR4	ACCAAGCAAAGAAAATTCTACC	*cox*1*-rrn*S^2^	Present study
3CF5	TGTTAAAAGGCTTTGGTGTG	*rrn*S*-nad*5^2^	Present study
3CR5-1	ACCAACCAAACCTACACATC	*rrn*S*-nad*5^2^	Present study
3CF6-1	TTACGTTAGTTGGGTTGTTG	*nad*5*-cox*3^2^	Present study
3CR6	TTACATCTTTATAAAACACTTTC	*nad*5*-cox*3^2^	Present study

^1^ short regions amplified by PCR from *cox*3 (139 bp), *cyt*b (613 bp), *nad*4 (554 bp), *cox*1 (497 bp), *rrn*S (500 bp) and *nad*5(458 bp). ^2^ large fragments that were amplified by long-range PCR from *cox*3-*cyt*b (724 bp), *cyt*b-*nad*4 (1008 bp), *nad*4-*cox*1 (4675 bp), *cox*1-*rrn*S (2198 bp), *rrn*S-*nad*5 (1981 bp) and *nad*5-*cox*3 (1718 bp)

Then, 12 additional primers (Table 1) were designed based on the obtained sequencing results to amplify six regions from genomic DNA (~40-80 ng) by long-PCR. PCRs (50 μl) were performed in reactions containing 0.4 mM each of dNTPs, 5 μl 10× LA Taq buffer II(Mg^2+^ Plus), 2.5 μM of each primer, 2.5 U LA *Taq* polymerase (Takara) and 2.5 μl genomic DNA. And the reactions were run under the following program: 94 °C for 5 min, followed by 35 cycles of 94 °C/30 s, 50 °C/30 s and 72 °C/1-5 min (depending on the size of *F. hepatica*). Amplicons were cloned into pGEM-T-Easy vector (Promega, USA) and then sequenced using a primer-walking strategy [18].

### Sequence analyses

*F. elongates* mt genome sequences were assembled manually and then aligned with the mt genome sequences of *F. hepatica*, *C. sinensis* and *P. cervi* using the program Clustal X 1.83 [19]. Open reading frames were identified by ORF Finder (http://www.ncbi.nlm.nih.gov/gorf/gorf.html) using the echinoderm and flatworm mitochondrial code. Initiation and termination codons of the 12 protein-coding genes were identified as reported [15]. The 22 tRNA genes were predicted using tRNAscan-SE or manual adjustments [20,21]. The two rRNA genes were predicted by comparison with those of *F. hepatica* [15], *C. sinensis* [16] and *P. cervi* [17]. Amino acid sequences of 12 protein-coding genes were inferred using ExPASy Translate tool (http://web.expasy.org/translate/) using the echinoderm and flatworm mitochondrial codes, and aligned using MEGA 5.0 with default settings [22].

### Nucleotide variation analysis

The nucleotide variation between *F. elongates* and *P. cervi* was analysed by sliding window analysis as reported [17].

### Phylogenetic analysis

Amino acid sequences translated from individual genes of the mt genome of *F. elongates* were aligned with those predicted from mt genomes of selected trematodes, including *C. sinensis* (NC_012147) [16], *Dicrocoelium dendriticum* (NC_025280.1) [23], *F. hepatica* (NC_002546) [15], *Haplorchis taichui* (NC_022433.1) [24], *Metagonimus yokogawai* (KC330755.1), *Opisthorchis viverrini* (JF739555.1) [25], *P. cervi* (NC_023095.1) [17], *Schistosoma haematobium* (NC_008074) [26], *Schistosoma japonicum* (AF215860) [15], *Schistosoma mekongi* (NC_002529) [27], *Schistosoma spindale* (NC_008067) [26], and the cestode *Taenia solium* (outgroup) (NC_004022.1) [28]. The amino acid sequences of selected trematodes were aligned using MEGA 5.0 [22], and phylogenetic analysis of the aligned amino acid sequences was conducted in MEGA 5.0 using the Maximum Likelihood (ML) method.

## Results and discussion

### Features of the mt genome of *F. elongates*

The complete mitochondrial genome of *F. elongates* (GenBank accession no. KM_397348) is 14,120 bp in length. The length of the *F. elongates* mt genome is larger than the mtDNA genomes of *C. sinensis* (13,875 bp) and *S. japonicum* (14,085 bp), but smaller than *D. dendriticum* (14,884 bp), *F. hepatica* (14,462 bp), *H. taichui* (15,130 bp), *M. yokogawai* (15,258 bp), *S. haematobium* (15,003 bp), *S. mekongi* (14,072 bp) and *S. spindale* (16,901 bp).

**Fig. 1:**
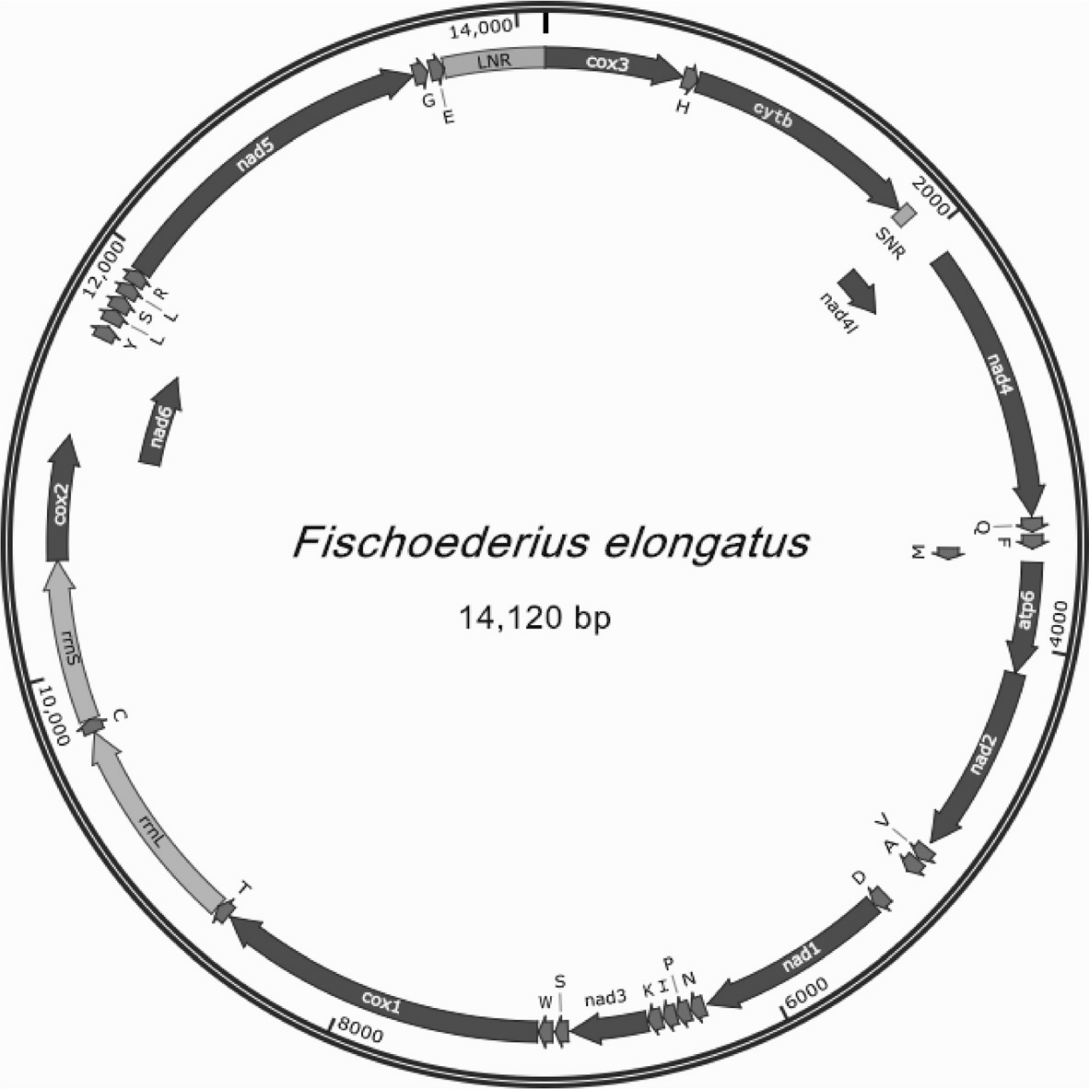
Arrangement of the mitochondrial genome of *Fischoederius elongatus*

The circular mt genome of *F. elongates* includes 12 protein-coding genes (*cox*1-3, *nad*1-6, *nad*4L, *cyt*b and *atp*6), 22 tRNA genes, two rRNA genes (*rrn*S and *rrn*L) and two non-coding regions (SNR and LNR). All the 12 protein-coding genes are transcribed in the same direction (Fig. 1), which is the same as in *F. hepatica* [15], *C. sinensis* [16] and *P. cervi* [17]. The gene arrangement order is as follow: *cox*3-*cyt*b-*nad*4L-*nad*4-*atp*6-*nad*2-*nad*1-*nad*3-*cox*1-*rrn*L-*rrn*S-*cox*2-*nad*6-*nad*5, which is consistent with *F. hepatica*, *O. viverrini*, *P. cervi*, *S. japonicum* and *S. mekongi*, except for *S. haematobium* and *S. spindale* [26].

Overlapping nucleotides between mt genes of *F. elongates* ranged from 1 to 53 bp (Table 2). The *F. elongates* mt genome has 26 intergenic spacers ranging from 1 bp to 148 bp in length (Table 2). The nucleotide contents of A, C, T and G in the mt genome are 19.78 %, 9.62 %, 44.10 % and 26.50 %, respectively (Table 3), with T being the most favored nucleotide, followed by G, A and C, which is also the same as the mt genomes of *F. hepatica* [15], *C. sinensis* [16] and *P. cervi* [17]. The A + T content of 12 protein coding genes and 22 rRNA genes of *F. elongates* ranged from 59.65 % (*rrn*S) to 66.97 % (*cox*3), and the overall A + T content of the mt genome is 63.88 %.

**Table 2:** The organization of the mitochondrial genome of Fischoederius elongatus

Gene/region	Positions	Size (bp)	Number of aa^1^	Ini/Ter codons^2^	Anticodons	In^3^
*cox*3	1-645	645	215	ATG/TAG		0
*trn*H	648-715	68			GTG	+2
*cyt*b	717-1829	1113	371	ATG/TAA		+1
SNR	1830-1892	63				0
*nad*4L	1893-2156	264	88	ATG/TAG		0
*nad*4	2117-3397	1281	427	GTG/TAA		−38
*trn*Q	3409-3471	63			TTG	+11
*trn*F	3486-3549	65			GAA	+14
*trn*M	3549-3612	64			CAT	−1
*atp*6	3613-4128	516	172	ATG/TAG		0
*nad*2	4133-5008	876	292	GTG/TAG		+4
*trn*V	5039-5102	64			TAC	+30
*trn*A	5109-5179	71			TGC	+6
*trn*D	5328-5397	70			GTC	+148
*nad*1	5400-6296	897	299	ATG/TAG		+2
*trn*N	6314-6379	66			GTT	+17
*trn*P	6384-6447	64			TGG	+4
*trn*I	6449-6511	63			GAT	+1
*trn*K	6518-6582	65			CTT	+6
*nad*3	6587-6943	357	119	ATG/TAG		+4
*trn*S1	6955-7014	60			GCT	+11
*trn*W	7027-7091	65			TCA	+12
*cox*1	7095-8636	1542	514	GTG/TAA		+3
*trn*T	8646-8709	64			TGT	+9
*rrn*L^4^	8710-9704	995				0
*trn*C	9707-9767	61			GCA	+2
*rrn*S^4^	9768-10518	751				0
*cox*2	10519-11100	582	194	ATG/TAG		0
*nad*6	11046-11546	501	167	ATG/TAG		−53
*trn*Y	11568-11632	65			GTA	+21
*trn*L1	11652-11715	64			TAG	+19
*trn*S2	11717-11785	69			TGA	+1
*trn*L2	11792-11856	65			TAA	+6
*trn*R	11860-11925	66			TCG	+3
*nad*5	11926-13506	1581	527	GTG/TAG		0
*trn*G	13510-13574	65			TCC	+3
*trn*E	13587-13651	65			TTC	+12
LNR	13652-14120	469				0

The inferred length of amino acid sequence of 12 protein-coding genes: ^1^amino acid; ^2^initiation and termination codons; ^3^intergenic nucleotides; ^4^initiation or termination positions of ribosomal RNAs defined by adjacent gene boundaries

**Table 3:** Nucleotide contents of genes and the non-coding region within the mitochondrial genome of Fischoederius elongatus

Gene	A(%)	C(%)	G(%)	T(%)	A + T(%)
*cox*3	18.29	8.53	24.50	48.68	66.97
*cyt*b	18.96	8.89	26.33	45.82	64.78
SNR	20.63	4.76	31.75	42.86	63.49
*nad*4L	21.97	8.33	25.38	44.32	66.29
*nad*4	16.55	9.52	25.45	48.48	65.03
*atp*6	17.64	10.08	24.42	47.87	65.50
*nad*2	15.64	7.99	25.11	51.26	66.89
*nad*1	16.39	7.47	28.21	47.94	64.33
*nad*3	15.97	7.84	28.01	48.18	64.15
*cox*1	18.87	11.02	24.51	45.59	64.46
*rrn*L	25.83	10.35	26.73	37.09	62.91
*rrn*S	24.37	12.25	28.10	35.29	59.65
*cox*2	19.93	11.11	27.49	41.58	61.51
*nad*6	17.44	8.61	26.71	47.24	64.68
*nad*5	16.32	8.29	28.78	46.62	62.93
LNR	26.01	9.17	26.44	38.38	64.39

The present *F. elongates* mt genome can provide useful information for the studies of epidemiology, species identification and genetic diversity of *Fischoederius spp*. At the same, it will also make contribution to the taxonomy study of *Fischoederius spp*. With the full mt genome of *F. elongates*, we can undertake a study within *F. elongates* from different regions or among *Fischoederius spp*. by combining the morphological features with genetic analyses (with molecular markers from mitochondria or ribosome, such as *cox*1, *nad*4, 18S, ITS-1 and ITS-2). Meanwhile, the mt genome of *F. elongates* may also provide information for the prevention and diagnosis of *Fischoederius spp*. and perhaps, this mt genome information may assist in the new drug, since mitochondria is the target of some drugs, such as decoquinate.

### Protein-coding genes

The *F. elongates* mt genome has 12 protein-coding genes, including *cox*3, *cyt*b, *nad*4L, *nad*4, *atp*6, *nad*2, *nad*1, *nad*3, *cox*1, *cox*2, *nad*6 and *nad*5. For these protein coding genes, ATG (eight of 12 protein genes) is the most common initiation codon, followed by GTG (four of 12 protein genes) (Table 2), which is the same as other trematodes, such as *F. hepatica* [15], *C. sinensis* [16], *P. cervi* [17], *S. mekongi* [27]. TAG (seven of 12 protein genes) or TAA (five of 12 protein genes) are the termination codons, this is in agreement with other digeneans, except for *P. cervi* (Only TAG was used as termination codons). Excluding the termination codons, 10,107 nucleotides encode 3,369 amino acids of protein-coding genes in the *F. elongates* mt genome. The most frequently used amino acid is TTT (Phe), with the frequency of 9.65 %, followed by TTT (Phe), TTG (Leu: 8.61 %), GTT (Val: 5.25 %) and TAT (Tyr: 5.02 %) (Table 4). The least used codons are AAC (Asn: 0.06 %), GAC (Asp: 0.06 %) and CGC (Arg: 0).

**Table 4:** Codon usage for 12 protein-coding genes in the mitochondrial genome of Fischoederius elongatus

Amino acid	Codon	Number	Frequency(%)	Amino acid	Codon	Number	Frequency(%)
Phe	TTT	325	9.65	Ile	ATT	127	3.77
Phe	TTC	28	0.83	Ile	ATC	6	0.18
Leu	TTA	167	4.96	Ile	ATA	71	2.11
Leu	TTG	290	8.61	Met	ATG	105	3.12
Ser	TCT	118	3.50	Met	GTG	165	4.90
Ser	TCC	6	0.18	Thr	ACT	54	1.60
Ser	TCA	22	0.65	Thr	ACC	3	0.09
Ser	TCG	25	0.74	Thr	ACA	19	0.56
Tyr	TAT	169	5.02	Thr	ACG	16	0.47
Tyr	TAC	11	0.33	Asn	AAT	54	1.60
Stop	TAA	3	0.09	Asn	AAC	2	0.06
Stop	TAG	9	0.27	Asn	AAA	23	0.68
Cys	TGT	112	3.32	Lys	AAG	50	1.48
Cys	TGC	9	0.27	Ser	AGT	92	2.73
Trp	TGA	41	1.22	Ser	AGC	9	0.27
Trp	TGG	72	2.14	Ser	AGA	31	0.92
Leu	CTT	43	1.28	Ser	AGG	35	1.04
Leu	CTC	3	0.09	Val	GTT	177	5.25
Leu	CTA	17	0.50	Val	GTC	12	0.36
Leu	CTG	23	0.68	Val	GTA	58	1.72
Pro	CCT	53	1.57	Ala	GCT	95	2.82
Pro	CCC	4	0.12	Ala	GCC	4	0.12
Pro	CCA	11	0.33	Ala	GCA	13	0.39
Pro	CCG	15	0.45	Ala	GCG	33	0.98
His	CAT	41	1.22	Asp	GAT	62	1.84
His	CAC	7	0.21	Asp	GAC	2	0.06
Gln	CAA	13	0.39	Glu	GAA	17	0.50
Gln	CAG	14	0.42	Glu	GAG	67	1.99
Arg	CGT	45	1.34	Gly	GGT	165	4.90
Arg	CGC	0	0	Gly	GGC	16	0.47
Arg	CGA	6	0.18	Gly	GGA	22	0.65
Arg	CGG	11	0.33	Gly	GGG	51	1.51

### Transfer RNA and ribosomal RNA genes

The *F. elongates* mt genome encodes 22 tRNAs, and the length of 22 tRNA genes ranged from 60 bp to 71 bp (Table 2). There are two non-coding regions in *F. elongates* mt genome, *rrn*S (751 bp) and *rrn*L (995 bp) (Table 2). The location of *rrn*S is between tRNA-Cys and *cox*2 and the *rrn*L is between tRNA-Thr and tRNA-Cys, which is the same as other trematodes, such as *F. hepatica* [15], *C. sinensis* [16] and *P. cervi* [17].

### Non-coding regions

Many flatworms have non-coding regions, it’s common to find two non-coding regions in trematodes: one long non-coding region (LNR) and one short non-coding region (SNR). In *F. elongates*, there is a short non-coding region (SNR: 62 nucleotides), which is located between *cyt*b and *nad*4L. In addition, there is also a long non-coding region (LNR: 468 nucleotides) between tRNA-Phe and *cox*3 (Table 2), the LNR has two obvious features, one is microsatellite-like sequences, such as (TA)n (n <5); the other is homopolymer sequences, such as (T)n (n <7). People still don’t understand clearly why the non-coding regions exist, and the function of them, people just knew the non-coding regions may participate in the replication of mitochondria [26].

### Nucleotide variability between *F. elongates* and *P. cervi*

**Fig. 2:**
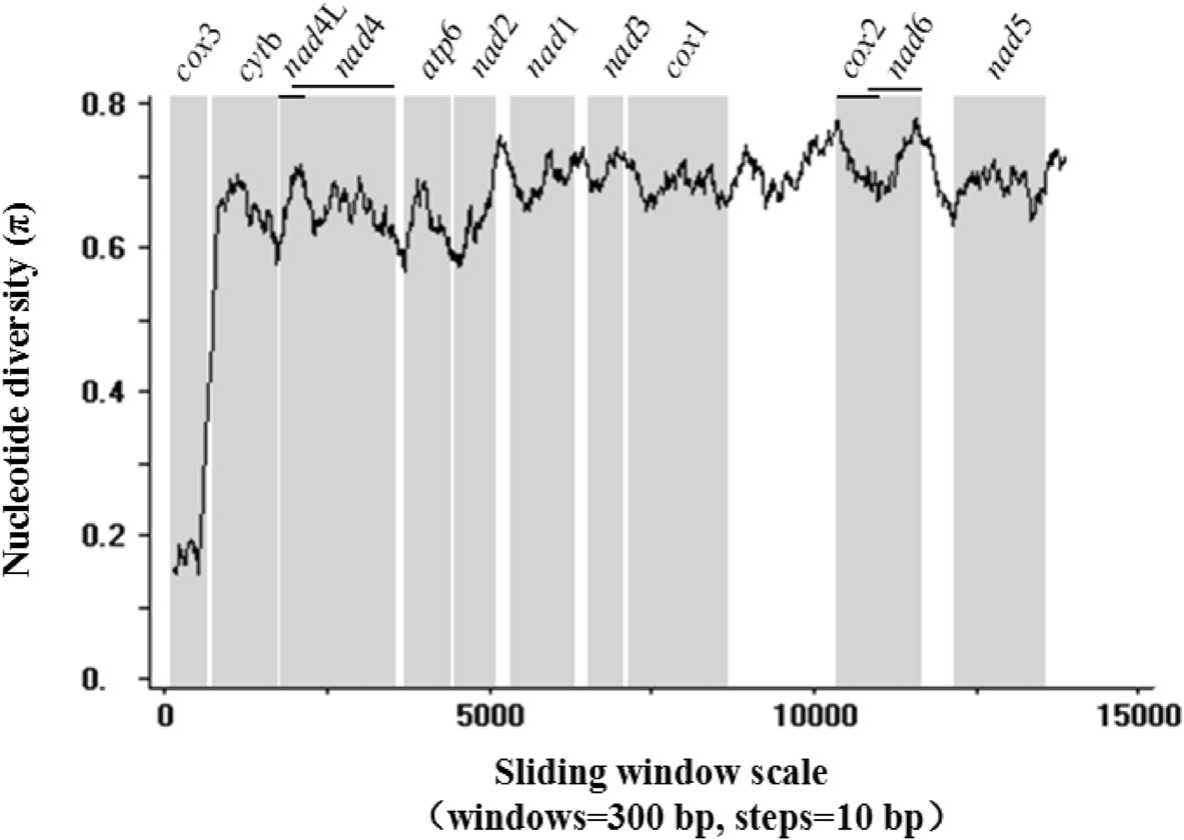
A sliding window analysis of complete mt genome sequences of *Fischoederius elongatus* and *Paramphistomum cervi.* The black line showed nucleotide diversity in a window of 300 bp (10 bp steps). *Nad*4L and *nad*4, *cox*2 and *nad*6 are overlapping genes. Gene regions are marked in grey boxes and boundaries are indicated

A sliding window analysis of *F. elongates* and *P. cervi* using full mt genome sequences reflected the nucleotide diversity (π) for all the protein-coding genes (Fig. 2). The highest and lowest level of nucleotide variability was within *nad*6 and *cox*3, respectively. In our study, *nad*6 and *cox*2 are the most conserved genes, and *cox*3 and *atp*6 are the least conserved. With sliding window analysis, we could know the conserved regions of mt genome among species.

### Genetic relationships

**Fig. 3:**
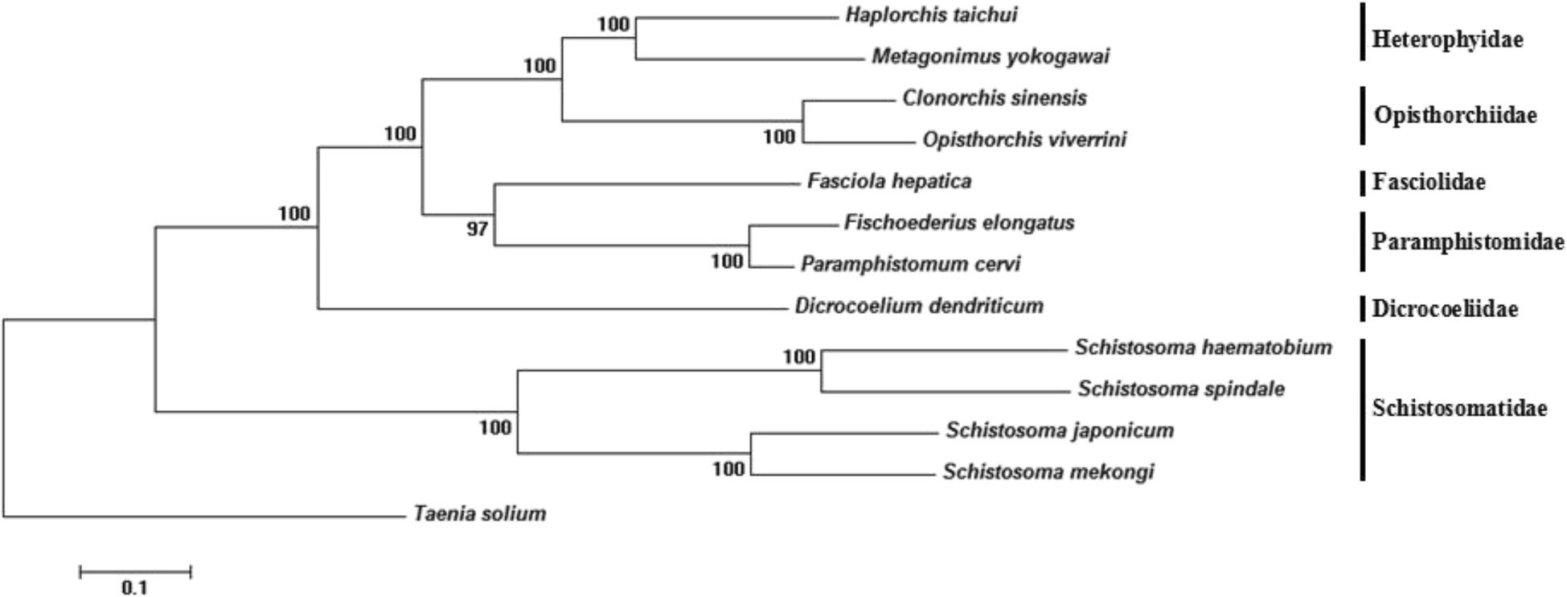
The phylogenetic relationships of *Fischoederius elongatus* and other trematodes based on concatenated amino acid sequence data representing 12 protein-coding genes by Maximum Likelihood analysis, using *Taenia solium* as an outgroup

Concatenated amino acid sequence data representing 12 protein-coding genes of 11 digenean species (*C. sinensis*, *D. dendriticum, F. hepatica*, *H. taichui*, *M. yokogawai*, *O. viverrini*, *P. cervi*, *S. haematobium*, *S. japonicum*, *S. mekongi* and *S. spindale*) and one tapeworm (*T. solium*) were used for genetic relationship analysis (Fig. 3). In the tree, we can find two large clades with strong support (100 %): one clade consists of eight members representing five families (Heterophyidae, Opisthorchiidae, Fasciolidae, Paramphistomidae and Dicrocoeliidae); the other clade is Schistosomatidae. In the present analysis, *F. elongates* has the closest genetic relationship with *P. cervi* (100 %), followed by Fasciolidae, this is consistent with their relationship in the classification of biology. At the same time, we also used NJ method analysis (not shown), and there was no difference between these two methods.
